# Thyroid cancer patients satisfaction at the management outcome: an analysis of the results of a nationwide survey in 485 subjects

**DOI:** 10.1186/s12913-021-06158-0

**Published:** 2021-02-18

**Authors:** Juan J. Díez, Juan C. Galofré

**Affiliations:** 1 Department of Endocrinology, Hospital Universitario Puerta de Hierro Majadahonda, Calle Manuel de Falla, 1, 28222 Majadahonda, Madrid, Spain; 2 Instituto de Investigación Sanitaria Puerta de Hierro Segovia de Arana (IDIPHISA), Madrid, Spain; 3 Department of Medicine, Universidad Autónoma de Madrid, Madrid, Spain; 4 Thyroid Task Force from the Sociedad Española de Endocrinología y Nutrición (SEEN), Madrid, Spain; 5 Department of Endocrinology, Clínica Universidad de Navarra, Pamplona, Spain; 6 Instituto de Investigación Sanitaria de Navarra (IdiSNA), Pamplona, Spain

**Keywords:** Satisfaction, Thyroid cancer, Healthcare services, Survey

## Abstract

**Background:**

We aimed to measure satisfaction of patients with thyroid cancer concerning different aspects of healthcare.

**Methods:**

We developed a web-based survey. Questions focused on patient satisfaction with specialists, the health centers and departments, and the information received about their disease. Level of satisfaction was quantified using a scale of 1 to 5. Values ≥4 were considered a high degree of satisfaction.

**Results:**

Four hundred eighty-five patients (aged 43.4 ± 9.9 yrs., 88% females) completed the survey. A high overall satisfaction with the specialists was reported by 52.5% of patients. The most highly valued specialists were surgeons, oncologists, and endocrinologists. 56.5% of respondents reported a high overall satisfaction with the health centers and departments. Lastly, the proportion of patients who were highly satisfied with the information received was only 42.5%. The presence of complications was indirectly related with satisfaction with specialists and information. Satisfaction with health centers and services was directly related with the level of education and inversely related to the time of evolution of the disease.

**Conclusion:**

Our results show a high degree of overall satisfaction of thyroid cancer patients. However, satisfaction can be improved in some areas, such with regards to the information provided to patients.

**Supplementary Information:**

The online version contains supplementary material available at 10.1186/s12913-021-06158-0.

## Background

The management of thyroid cancer has dramatically improved in recent years. The changes are mainly impacting the two extremes of the disease spectrum, i.e., stage I and stage IV patients. Thus, several recent recommendations are focused on avoiding overdiagnosis (as this leads to overtreatment), while others are centered on the development of better therapeutic options for those patients with radioactive refractory disease.

The multidisciplinary team plays a key role in this increasingly complex scenario. Management of cancer involves several healthcare professionals and the role of a multidisciplinary team is crucial to achieving high standards [1]. At the same time, there is a general feeling that the patient has a role to play in the process, although it is not clear how to best include him or her [2, 3]. A first step might be to determine how the patient perceives the level of quality of the assistance received from healthcare givers [4, 5]. However, despite the emergent position of the patient-centered medicine in thyroid cancer, little is known about their satisfaction with care management [6].

Our purpose was to assess the level of satisfaction of Spanish thyroid cancer patients with their healthcare specialists in a large representative sample of individuals. We designed a questionnaire that allowed us to receive feed-back from thyroid cancer patients about their degree of satisfaction as to (1) the quality of care from the different specialists involved in the management of their disease, (2) the performance of the health services, and (3) their satisfaction with information received throughout the process.

## Methods

### Scope of the study

The scope of the study included all patients with thyroid cancer of any type who were members or supporters of the Spanish Association of Thyroid Cancer (Asociación Española de Cáncer de Tiroides, AECAT). This is a population of people motivated and concerned about their disease and the improvement of care for patients who suffer from thyroid cancer. AECAT is a nationwide association with supporters in all geographic regions of Spain and with the ability to disseminate information on thyroid cancer throughout the country, and to gather the opinion of patients on different topics. AECAT is a partner of the Thyroid Cancer Alliance.

### Study design

We designed a survey for patients with thyroid cancer to be answered anonymously (supplementary material). The design was based on previous surveys of patient satisfaction in oncology [7]. Before general release, several patients who were members of the Board of Directors of AECAT vetted the survey. The survey was divided into two sections: (1) demographic and clinical questions (gender, age, community of residence, education level, type of cancer and extent of the disease at diagnosis, type of hospital, therapies used, chronic complications from therapies, and disease status at time of survey); and (2) questions about patient satisfaction with the specialists who they visited, the health centers and departments they attended, and the information the respondents received about their illness, therapies and complications (supplementary material).

The strategy for creating the survey questions was designed to anonymize each interviewee. It was designed to be answered in no more than 10–15 min. Many questions were multiple choice with the possibility of choosing one or more of the answers. To avoid bias in the multiple-choice questions, attempts were made to avoid phrases that might elicit a particular response. A wide range of options arranged alphabetically or randomly was included.

### Inquired features of the disease status

To evaluate disease status, we asked about the tumor type, the tumor extension at diagnosis and the need for radioiodine treatment after surgery. We calculated the time of evolution of the disease from the date of surgery to the date of the study. To estimate the current status of the disease, we inquired about the medications currently being used and chronic complications arising from thyroid cancer or its treatment. We defined chronic as not temporary complications after the immediate surgery that last permanently. To estimate the disease situation at the time of the survey, we asked respondents to classify their current situation into one of  three categories: cured, not cured, but without need of therapy for their thyroid cancer (other than substitute treatment), and not cured, but in need of additional therapy for their cancer, either medical or surgical.

In the second part of the survey, we explored patient satisfaction in the three areas of interest by asking questions about 10 items in each area. The satisfaction for each item was scored from 1 to 5, with 1 being lowest satisfaction and 5 the highest. When enquiring about the surgeon, it was understood that they were specialists in general surgery or otolaryngology, since these are the two specialties that deal with thyroid surgery in Spain. Patients were asked to respond only to questions about specialists, healthcare services or information they needed or wanted at any point throughout the course of their illness. Since some patients did not have contact with some specialists or health services (e.g., radiotherapists, oncologists, psychologists, pathologists), we assumed that some questions would not apply and would not be answered. To assess overall satisfaction in each area of interest (specialists, health centers and information) in every patient, the arithmetic mean of the 10 evaluated items was calculated. When this overall satisfaction was greater than or equal to 4, it was considered high overall satisfaction.

### Contact with potential interview patients

All potential interviewees, that is, thyroid cancer patients who are partners or supporters of AECAT, received information from our survey through the association’s website (https://www.aecat.net) and through its patient forums. The authors did not establish or maintain correspondence with the potential study participants. The link giving access to the survey remained on the web from June 13 to October 6, 2019.

### Data collection

Participants’ responses were collected anonymously and stored electronically in a form hosted on an open access form creation website (https://www.google.es/intl/es/forms/about/). Data were accessible only to the authors of the survey and were password protected. Once the deadline for collecting surveys had passed, the data were gathered onto an electronic database for subsequent statistical processing.

### Ethical issues

This study was conducted with the endorsement of the Board of Directors of AECAT. It was also approved by the Clinical Research Ethics Committees of the Hospital Universitario Puerta de Hierro Majadahonda (Madrid) and the Clínica Universidad de Navarra (Pamplona). Written informed consent was waived by our institutional review boards because this study consisted of conducting a voluntary web-based survey. To maintain the anonymity of the participants, they were not asked their address, city, telephone number or any other personal data that could make possible their identification.

### Statistical analysis

For quantitative variables, results are expressed as mean ± SD for normally distributed data and as median (interquartile range, IQR) for nonparametric data. Adjustment to normal distribution was tested by the Kolmogorov-Smirnov test. Categorical variables are described as ratios or percentages. For comparisons of means between two groups of patients, the Student’s *t-*test was used for normally distributed data, and the Mann-Whitney U test was used for nonparametric data. For ratio comparisons, the chi-square test or Fisher’s exact test was used. Logistic regression analysis was used to assess the influence of putative predictive factors influencing the degree of overall satisfaction of patients with specialists, health services and received information. Two-sided tests were used, and differences were considered significant when *P* < 0.05.

## Results

### Surveyed patients

This web-based survey was completed by 486 patients. One patient was excluded because she suffered from parathyroid, but not thyroid cancer. The final analyzed data set consisted of completed questionnaires from 485 patients. Mean (±SD) age of patients responding to the survey was 43.4 ± 9.9 years, with a median (IQR) time of evolution of their disease of 4 (2–7) years. Most of the participants were females (88%) and suffered from papillary thyroid carcinoma (79%). Details of the demographic and clinical characteristics of these patients are provided in Table 1. The surveyed women were younger (*P* = 0.025) and with lower age at diagnosis (*P* = 0.015) than men, with similar evolution time of the disease (*P* = 0.703). In addition, women presented a lower percentage of treatments with tyrosine-kinase inhibitors (*P* = 0.007), and a higher cure rate than men (*P* = 0.007). No other significant differences were found between female and male patients.

**Table 1:** Demographic and clinical features of 485 surveyed patients with thyroid cancer

	All (***n*** = 485)	Females (***n*** = 429)	Males (***n*** = 56)
Age, yr	43.4 ± 9.9	43.0 ± 9.7	46.4 ± 10.6*
Age at diagnosis, yr	37.5 ± 10.3	37.1 ± 10.1	41.0 ± 11.0*
Time of evolution, yr	4 (2–7)	4 (2–8)	4 (2–7)
Education			
Primary or secondary	244 (50.3)	216 (50.3)	28 (50.0)
University	241 (49.7)	213 (49.7)	28 (50.0)
Tumor type			
Papillary	382 (78.8)	338 (78.8)	44 (78.6)
Follicular	40 (8.2)	37 (8.6)	3 (5.4)
Medullary	27 (5.6)	21 (4.9)	6 (10.7)
Other	18 (3.7)	17 (4.0)	1 (1.8)
Unknown	18 (3.7)	16 (3.7)	2 (3.6)
Tumor extension at diagnosis			
Thyroid	247 (50.9)	221 (51.5)	26 (46.4)
Thyroid and lymph nodes	206 (42.5)	181 (42.2)	25 (44.6)
Distant metastases	23 (4.7)	19 (4.4)	4 (7.1)
Unknown	9 (1.9)	8 (1.9)	1 (1.8)
Type of hospital			
Private	81 (16.7)	67 (15.6)	14 (25.0)
Public	367 (75.7)	328 (76.5)	39 (69.6)
Both	37 (7.6)	34 (7.9)	3 (5.4)
Radioiodine			
No	91 (18.8)	79 (18.4)	12 (21.4)
1 dose	269 (55.5)	245 (57.1)	24 (42.9)
2 doses	92 (19.0)	78 (18.2)	14 (25.0)
More than 2 doses	31 (6.4)	25 (5.8)	6 (10.7)
Unknown	2 (0.4)	2 (0.5)	0 (0)
Medications			
Levothyroxine	481 (99.2)	425 (99.1)	56 (100)
Calcium salts	150 (30.9)	138 (32.2)	12 (21.4)
Active vitamin D	81 (16.7)	75 (17.5)	6 (10.7)
Tyrosine-kinase inhibitors	12 (2.5)	7 (1.6)	5 (8.9)**
Disease situation at the time of survey			
Cured	252 (52.0)	233 (54.3)	19 (33.9)**
Not cured, no need of therapy	175 (36.1)	150 (35.0)	25 (44.6)
Not cured, in need of therapy	58 (12.0)	46 (10.7)	12 (21.4)
Chronic complications			
Local discomfort or pain	186 (38.4)	158 (36.8)	28 (50.0)
Hypoparathyroidism	152 (31.3)	140 (32.6)	12 (21.4)
Dry mouth	148 (30.5)	132 (30.8)	16 (28.6)
Dysphonia	146 (30.1)	123 (28.7)	23 (41.1)
Neck mobility problems	92 (19.0)	80 (18.6)	12 (21.4)
Dysgeusia	62 (12.8)	58 (13.5)	4 (7.1)
Other	156 (32.2)	134 (31.2)	22 (39.3)

Data are the mean ± SD or the median (interquartile range) for quantitative variables, and the number (percentage) for categorical variables

*P < 0.05; **P < 0.01

### Geographical distribution and use of health resources

**Fig. 1:**
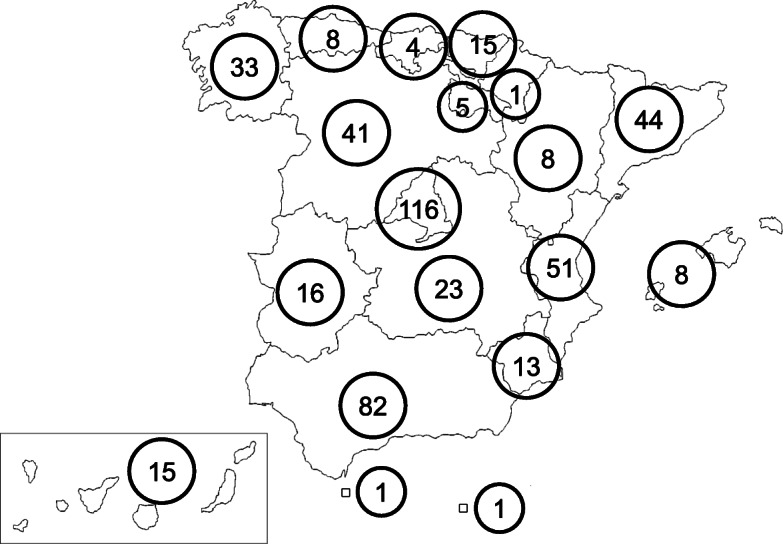
Geographic distribution of patients with thyroid cancer who responded to the survey. The blank map was freely available at the website: https://commons.wikimedia.org/wiki/File:CCAA_of_Spain_(Blank_map).PNG

Figure 1 shows the geographical distribution of respondents throughout the 17 autonomous regions of Spain. As expected, larger number of respondents came from most populated regions, i.e., Madrid (pop. 6,578,079), Andalusia (pop. 8,384,408), Valencia (pop. 4,963,703), and Catalonia (pop. 7,600,065) [8].

The majority of patients utilized the public healthcare system for the control and monitoring of their disease, although 81 patients (16.7%) used exclusively private healthcare and 37 (7.6%) combined public and private healthcare services. The specialist typically relied on for thyroid cancer follow-up visits was the endocrinologist in 421 patients (86.8%). The remaining patients had as their usual physician specialists in general surgery-otolaryngology in 27 cases (5.6%), medical oncology in 21 cases (4.3%), family medicine in 14 cases (2.9%) and radiotherapy in 2 cases (0.4%).

### Satisfaction scores

In the assessment of specialists, the highest percentages of high satisfaction were awarded to surgeons (i.e., specialists in general surgery or otolaryngology) (69.1%, 452 respondents), oncologists (64.3%, 42 respondents) and endocrinologists (64.0%, 467 respondents). The lowest percentages of satisfaction were with pathologists (35.2%, 213 respondents), general practitioners (30.1%, 369 respondents) and radiologists (28.8%, 250 respondents). The mean overall satisfaction with the specialists was 3.68 ± 1.15 over 5. However, 52.5% of patients indicated a high (score ≥ 4) overall satisfaction with their specialists.

**Fig. 2:**
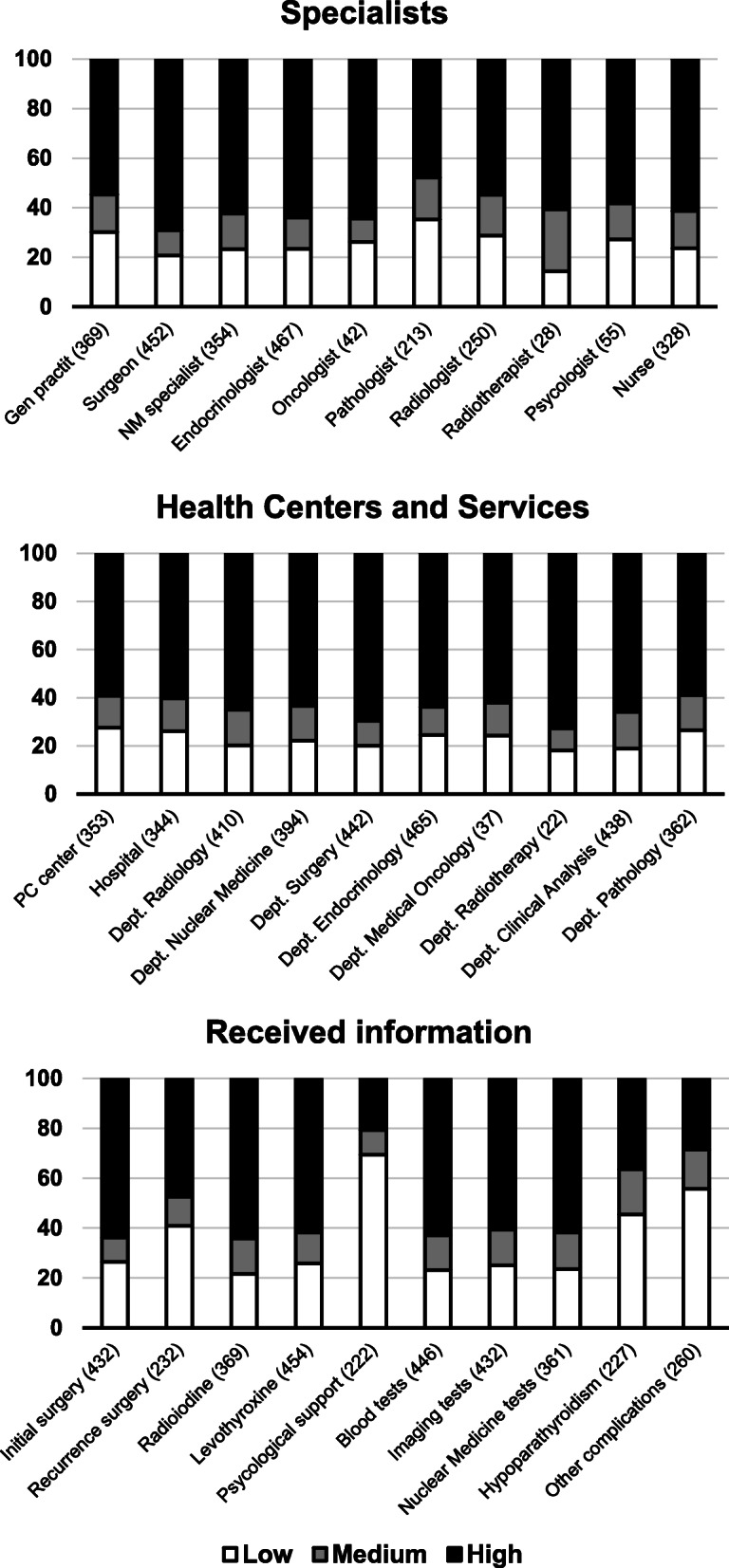
Satisfaction scores with specialists, health centers and services and received information about the disease and its treatments. Patients provided their appreciation of satisfaction with the different requested issues at each area of interest, scoring from 1 (lower satisfaction) to 5 (higher satisfaction). Each rectangle indicates the percentage of high (4–5, black), medium (3, gray) and low (1–2, white) satisfaction. Numbers in brackets indicate the number of responses obtained in each item. Abbreviations: MN, nuclear medicine; PC, primary care

The most highly rated health services were the departments of radiotherapy (72.7% of the patients with high satisfaction, 22 respondents), surgery (69.5% high satisfaction, 442 respondents) and clinical analysis (65.8% high satisfaction, 438 respondents). The worst rated were the primary care center, the department of pathology and the hospital outpatient clinic, with low satisfaction (score ≤ 2) rates of 27.5, 26.5 and 26.1%, respectively. A high overall satisfaction was reported by 56.5% of patients (Fig. 2).

Patients were highly satisfied with the information they received about radioiodine treatment, initial surgery, and blood tests, with high percentages of satisfaction of 64.2% (369 respondents), 63.9% (432 respondents) and 63.0% (446 respondents), respectively. Responding patients showed a high degree of dissatisfaction (score ≤ 2) with the information received about psychological support (69.4% low satisfaction, 222 respondents), treatment of other complications (55.7% low satisfaction, 260 respondents) and treatment of hypoparathyroidism (45.4% low satisfaction, 227 respondents). Overall satisfaction with information was 3.47 ± 1.22. High overall satisfaction with information was reported by 42.5% of responding patients (Fig. 2).

### Logistic regression analysis

Several models of logistic regression analysis were run to study the dependence of the variables related to satisfaction as a function of several independent variables (Table 2). Satisfaction with specialists was negatively related to complications of therapy (odds ratio [OR], 0.59; 95% confidence interval [CI], 0.36–0.95; *P* = 0.031). When analyzing satisfaction with health centers and services we found a positive association with the level of education (OR, 1.70, 95% CI 1.14–2.53; *P* = 0.009) and a negative association with the time of evolution of the disease (OR, 0.96; 95% CI, 0.93–0.99; *P* = 0.043). Lastly, we found that satisfaction with the received information was negatively related to complications of treatments (OR 0.53; 95% CI, 0.33–0.85; *P* = 0.008). No other significant relationships were found with the independent variables listed on Table 2.

**Table 2:** Results of three models of logistic regression to study the influence of several covariates on the presence of high satisfaction (score ≥ 4) in the three studied areas of interest (specialists, health centers and services and received information)

	Satisfaction with specialists			Satisfaction with health centers and services			Satisfaction with received information		
	OR	95% CI	***P***	OR	95% CI	***P***	OR	95% CI	***P***
Sex, male	1.71	0.92–3.17	0.088	1.58	0.83–3.00	0.162	1.11	0.61–2.01	0.738
Age, yr	1.01	0.99–1.03	0.519	1.02	0.99–1.04	0.066	1.01	0.99–1.03	0.385
Education, university	1.34	0.91–1.98	0.142	**1.70**	**1.14–2.53**	**0.009**	0.96	0.65–1.42	0.828
Tumor type, non-CDT	0.75	0.36–1.60	0.754	0.60	0.28–1.28	0.187	0.78	0.36–1.70	0.537
Tumor extension									
Thyroid	1			1			1		
Thyroid and lymph nodes	1.31	0.87–1.98	0.190	1.08	0.71–1.63	0.727	1.12	0.74–1.68	0.593
Distant metastases	1.25	0.46–3.34	0.663	1.15	0.43–3.11	0.784	1.01	0.37–2.76	0.984
Time of evolution, yr	0.98	0.94–1.01	0.154	**0.96**	**0.93–0.99**	**0.043**	0.98	0.94–1.01	0.154
Type of hospital, private	0.67	0.43–1.06	0.085	1.14	0.72–1.82	0.574	1.20	0.77–1.88	0.425
Radioiodine	0.93	0.53–1.66	0.816	0.83	0.46–1.50	0.546	0.99	0.55–1.76	0.968
Disease situation, not cured	0.66	0.35–1.26	0.208	0.72	0.38–1.37	0.321	0.91	0.48–1.74	0.914
Complications of therapy	**0.59**	**0.36–0.95**	**0.031**	0.61	0.37–1.01	0.052	**0.53**	**0.33–0.85**	**0.008**

Statistically significant values are indicated in bold

*Abbreviations*: *OR* odds ratio, *CI* confidence interval

## Discussion

Patient-reported quality of care, which is normally measured by patients’ overall rating of care, is gaining more attention within the area of oncology [2, 4, 5, 9, 10]. A survey of patient satisfaction is an important tool in determining the quality of healthcare. However, the concept of patient satisfaction is not very straightforward. It has been described as what is achieved when a patient’s treatment expectations are met or exceeded [11]. The effect on quality of life is primarily related to emotional and social impacts of treatment [12]. In addition, patient satisfaction depends on multiple factors not well categorized [5]. Satisfaction is related to experiences (fears, predictions), subject’s characteristics (age, sex, level of education, self-perceived health status) and external elements such as health-care structures (physical hospitals settings, cleanliness, comfort) personnel feed-back, processes (waiting time, coordination, information) and, obviously, disease management and outcomes [3]. Measuring satisfaction is important because it influences disease response to therapy [13]. Satisfied patients report higher quality of life and are more likely to comply with treatment, which may lead to better clinical results [14]. In this setting it is essential to identify the expectations that are and are not being met as this information may help to improve treatment policy and practices.

The management of differentiated thyroid cancer is the responsibility of a multidisciplinary team [1, 2]. There is consensus that patients have to form part of the multidisciplinary team. Not only is this difficult to achieve but doctors have not been trained for this [15]. In order to bridge the current gap between what is and could be, as a first step, patients would need to receive full verbal and written information about their condition, their treatment and have permanent access to a member of the multidisciplinary team for guidance and support [2]. Good quality information and patient involvement in decision-making processes are generally appreciated by most people while ambiguity and lack of information fuels anxiety and impacts negatively on satisfaction [2]. A lack of confidence in their doctor is an important obstacle to individuals seeking appropriate and timely assistance [13]. Thus, healthcare givers need to exhibit empathy and compassion.

Despite thyroid cancer has a good general prognosis, concern about the future is high among patients [2, 16–18]. A Swedish study reported that around 50% the patients continued to worry about possible recurrences, even 15 years after diagnosis [19]. Increased physician awareness of the detrimental effects of a thyroid cancer diagnosis on quality of life should allow for a more accurate conversation about expected outcomes following thyroid cancer treatment [20]. It has been reported that both surgeons and clinicians underestimate the percentage of thyroid cancer patients with anxiety, disease, or treatment related symptoms [21]. Symptoms such as temporary or permanent voice change (even in the absence of vocal cord palsy), temporary dry mouth (up to 80% in some reports), hot/cold sensitivity, and temporary and permanent hypocalcemia are not rare [21].

Our results support these findings and reveal the real-world situation in Spain where, to our knowledge, no such information has yet been published to date. Our sample is representative as it covers the entire country. Patient demographics indicate a representative distribution in terms of age and sex with percentage of thyroid cancer subtypes and outcomes akin to other similar studies. Not surprisingly, we find that few men are diagnosed, they are older than women, and have the worst prognosis.

In our data what is especially relevant is the received information. This help to shed light on the unreported treatment-related thyroid cancer events in Spain. In our survey, almost a third of the respondents had some discomfort related to the therapy. This is happening in an otherwise generally indolent disease. The high prevalence of reported chronic complications may be accounted for, at least in part, by the fact that patients with more aftermath or more troublesome sequelae are more likely to seek help from patient associations such as AECAT, which offer information services and help to their partners. The information could favor the use of less interventionist strategies [22] and emphasize the need to include the patient as an active member of the multidisciplinary team.

Interestingly, although respondents showed a high degree of satisfaction with information about initial surgery, they expressed a high level of dissatisfaction with information on the treatment of hypoparathyroidism and other complications. This probably reflects that doctors rarely explain the potential negative impact of hypoparathyroidism on quality of life. It is striking that the percentage of hypoparathyroidism is exceedingly high (31%), almost twofold higher than that found in a recent nationwide report [23]. This high prevalence of hypoparathyroidism may be related to the high degree of dissatisfaction, since patients with chronic hypoparathyroidism are especially sensitive to the sequelae of treatment. Our results agree with a recent study [24] showing that patients with permanent hypoparathyroidism strongly agree with the assertions “I am concerned with the long-term complications of my hypoparathyroidism medications” (75%), and “Hypoparathyroidism is harder to control and deal with compared to what the physician initially told me to expect “ (63%). Another study also stablished that patients with hypoparathyroidism reported significantly worse outcomes on quality of life variables, including overall health, ability to pursue social activities, paresthesias, muscle cramping, and medication interference with daily life compared to surgeons and preoperative controls [25].

On the other hand, more than 80% of participants received additional radioiodine therapy that could lead to a variety of side effects such dry mouth (30.5%) and dysgeusia (12.8%). Wallner et al. [26] have reported that the majority of patients who receive radioiodine perceived they did not have a choice about whether to receive the treatment or not. In general, patients are not normally included in shared and informed decision making about the necessity of complementing treatment with radioactive iodine [3]. Taken together, these results give strong support to the idea that shared decision making may provide an opportunity to reduce overtreatment and to ensure patient participation in the decision-making process.

As has been observed by others [5], highly educated people tend to be more satisfied with their healthcare personnel. This could be tied to their greater level of the understanding of the disease. And it is relevant that doctors dealing with thyroid cancer patients in Spain (and probably elsewhere) should improve the level of information they share with their patients.

This study has its limitations, just as all surveys. Results cannot be extrapolated to countries other than Spain. It is possible that AECAT member patients have a greater awareness of the disease and, hence, are more motivated than the overall population of patients with thyroid cancer in Spain. Besides, the self-perception of not cured by the patient can be biased as patients may not have a great knowledge of medical terms. Our study did not inquire about the extent of the initial surgery, although we can assume that most of them were treated with total thyroidectomy, since lobectomy has not been widely used in our country [27, 28]. The sample size of the satisfaction assessment of some specialties and departments was low, i.e., oncology, radiotherapy. This was because patients were asked to rate only those specialists and departments with whom they had had contact. It is possible that the low reported satisfaction with pathologists is influenced by a lack of exact knowledge of the work of this specialty. Results are based on the reports submitted by participating patients without independent verification. We understand that patients have been honest in their answers, although we cannot rule out that some may have exaggerated their symptoms or number of complications. This study was approved by the AECAT board of directors, which is the owner of the website where the survey was hosted. There were no legal claims during or after the study period. To ensure that results are reported as fully and accurately as possible we followed the STROBE guidelines (https://www.strobe-statement.org/index.php?id=strobe-home).

## Conclusion

In summary, satisfaction is challenging to objectify because it is related to various emotions, experiences, and situations. Still, the measurement of satisfaction in thyroid cancer, a frequent cancer, seems to be a necessary study in terms of treatment response. This study demonstrated for the first time that thyroid cancer patient self-reported satisfaction with healthcare providers is adequate in Spain, despite the high proportion of treatment-related side effects and the frequent complaints about the lack of information. These results underline the need to tailor the level of therapy to actual treatment needed within a multidisciplinary team where patients are represented as full members.

## Supplementary Information

**Additional file 1.**

## Data Availability

Data are available through a reasonable request to the corresponding author.

## Abbreviations

AECAT: Asociación Española de Cáncer de Tiroides (Spanish Association of Thyroid Cancer)
CI: Confidence interval
IQR: Interquartile range
NM: Nuclear medicine
OR: Odds ratio
PC: Primary care
SD: Standard deviation
STROBE: Strengthening the Reporting of Observational studies in Epidemiology
