# Supplementary material for: Thyroid cancer patients satisfaction at the management outcome: an analysis of the results of a nationwide survey in 485 subjects
Source: BMC Health Serv Res. 2021 Feb 18;21:158. doi: 10.1186/s12913-021-06158-0 (PMC7890898; doi:10.1186/s12913-021-06158-0)
Supplement: Supplementary file 1 — Additional file 1. [file 12913_2021_6158_MOESM1_ESM.doc]

**Authors**

**Juan J. Díez^1,2,3,4^ and Juan C. Galofré^4,5,6^**

**Tittle**

**Thyroid Cancer Patients Satisfaction at the Management Outcome: An Analysis of the Results of a Nationwide Survey in 485 Subjects**

**SUPPLEMENTARY MATERIAL**

English version of:

- Information and welcoming to the survey
- Instructions to fulfill the survey
- Survey for patients with thyroid cancer

**INFORMATION AND WELLCOMING TO THE SURVEY**

Dear friend,

We invite you to participate in a survey, which pretends to know your opinion about some aspects of the healthcare that you have received as patient with thyroid cancer.

The survey has been designed by Dr Díez and Dr Galofré, endocrinologists of the hospitals Puerta de Hierro Majadahonda and Clínica Universidad de Navarra, respectively. The objective of the survey is knowing the opinion of thyroid cancer patients about their satisfaction with the attention received, and the perception of the way of working of the medical centres responsible for their treatment.

The authors of the survey have received the approval of the AECAT (Asocición Española de Cáncer de Tiroides, Spanish Association of Thyroid Cancer) Board of Directors for its dissemination in the website of the association. The survey is anonymous and the authors will not collect any data that could enable the identification of the participants.

The aim of the study is exclusively to obtain and register an information of which there are no existing data in this moment in Spain. Once the study has been finalized, the results will be reported to: (1) the members of AECAT, (2) the scientific community through its presentation in scientific meetings and publications, and (3) the health authorities that could be interested in the care of patients with thyroid cancer.

There are no financial purposes in this study, nor have the authors received any fee or remuneration from any public or private entity for the design or execution of the study.

The AECAT Board of Directors encourages you to participate since your opinion will be very useful to have real data on the opinion of patients, which can be used to improve care for patients with thyroid cancer.

We acknowledge and thank you for your participation, and wish you all the best,

JUNTA DIRECTIVA DE AECAT

AECAT BOARD OF DIRECTORS

**INSTRUCTIONS TO FULFILL THIS SURVEY**

The following survey is divided in two sections. In the first one, we request your demographic and clinical data. In the second part, we request your opinion about some aspects of the healthcare received and satisfaction level.

The survey is anonymous, and your participation is voluntary.

Most questions can be answered by an X in the appropriate option. Others with a number. The degree of satisfaction is answered with a number between 1 (minimum satisfaction) and 5 (maximum satisfaction). The survey is estimated to take 10-15 minutes.

The questionnaire is to be completed online and the data remains anonymous for the sole purpose of informing partners, academics and scientists.

The Asociación Española de Cáncer de Tiroides (AECAT) thanks you in advance for your participation in this survey.

JUNTA DIRECTIVA DE AECAT

AECAT BOARD OF DIRECTORS

**SURVEY FOR PATIENTS WITH THYROID CANCER**

**1. DEMOGRAPHIC AND CLINICAL DATA**

**Question 1**

Sex

|  | Female |
| --- | --- |
|  | Man |

**Question 2**

Current age (years)

| Numerical answer |
| --- |

**Question 3**

Region of residence (select one)

|  | Andalucía |
| --- | --- |
|  | Aragón |
|  | Asturias |
|  | Islas Baleares |
|  | Canarias |
|  | Cantabria |
|  | Castilla-La Mancha |
|  | Castilla y León |
|  | Cataluña |
|  | Comunidad Valenciana |
|  | Extremadura |
|  | Galicia |
|  | La Rioja |
|  | Comunidad de Madrid |
|  | Región de Murcia |
|  | Comunidad Foral de Navarra |
|  | País Vasco |
|  | Ceuta |
|  | Melilla |

**Question 4**

Academic level

|  | No studies |
| --- | --- |
|  | Primary studies |
|  | Secondary studies |
|  | University studies |

**Question 5**

Type of thyroid cancer (select one)

|  | Papillary |
| --- | --- |
|  | Follicular |
|  | Medullary |
|  | Other |
|  | I don’t know |

**Question 6**

Thyroid cancer extension at diagnosis (select one)

|  | Limited to thyroid gland |
| --- | --- |
|  | Thyroid gland and neck lymph nodes |
|  | Extension to other organs beyond the neck (lung, bone, or other organs metastases) |
|  | I don’t know |

**Question 7**

Age at diagnosis (years)

| Numerical answer |
| --- |

**Question 8**

Have you received treatment with radioactive iodine admitted to a hospital? (iodine scintiscans that do not require hospital admission are here excluded)

|  | No, never |
| --- | --- |
|  | One time |
|  | Two times |
|  | More than two times |
|  | I don’t know |

**Question 9**

If you have received therapy with radioactive iodine, how was the preparation to receive this therapy?

|  | I was always advised not to take thyroid hormone during the previous month |
| --- | --- |
|  | With recombinant TSH injections (Thyrogen), so I did not have to stop thyroid hormone administration |
|  | Both previous ways |
|  | I don’t know |
|  | Not applicable, I have not received radioactive iodine treatment |

**Question 10**

Year of the last dose of radioactive iodine

| Numerical answer |
| --- |

**Question 11**

Indicate if you have or not the following aftermath of thyroid cancer treatment permanently (random order)

| No | Yes |  |
| --- | --- | --- |
|  |  | Dysphonia or aphonia |
|  |  | Hypoparathyroidism (need to take calcium and vitamin D) |
|  |  | Paralysis of a vocal cord |
|  |  | Lack of taste sensation |
|  |  | Dry mouth or lack of saliva |
|  |  | Pain or tenderness in the scar area |
|  |  | Mobility problems in the neck |
|  |  | Other chronic sequelae |

**Question 12**

Indicate whether or not you take any of the following medications related to thyroid cancer

| No | Yes |  |
| --- | --- | --- |
|  |  | Thyroid hormone (Eutirox, Levothroid, Levotiroxina) |
|  |  | Calcium |
|  |  | Rocaltrol (calcitriol) |
|  |  | Specific medication for cancer (tyrosine-kinase inhibitors) |

**Question 13**

Clinical status at the present time

|  | I am cured, I only do visits with blood tests and ultrasounds |
| --- | --- |
|  | I am not fully cured. I need check-ups with my doctor, but I don't need any surgery or radioactive iodine or cancer-specific medication for now |
|  | I am not cured and I need specific cancer therapy (surgery, radioactive iodine, cancer-specific medication) |

**Question 14**

How often do you forget to take your thyroid hormone pills?

|  | I take my pill rigorously every day, I never forget it |
| --- | --- |
|  | I forget my pill occasionally (less than 4 times a month) |
|  | I forget my pill often (5 or more times a month) |

**Question 15**

Where do your thyroid cancer follow-up visits take place?

|  | Private health centre |
| --- | --- |
|  | Public health centre |
|  | Both |

**2. OPINION SURVEY**

**Question 16**

Out of the following professionals that have or could have participate in the therapy or follow-up of your thyroid cancer, with which have you had personal contact? (select one or more)

(random order)

|  | Family doctor |
| --- | --- |
|  | Neck surgeon (either specialist in General Surgery or Otolaryngology) |
|  | Specialist in Nuclear Medicine (who administrates radioactive iodine) |
|  | Endocrinologist |
|  | Oncologist |
|  | Specialist in Pathology |
|  | Radiologist (who does neck ultrasound, CT scans, MRI, etc.) |
|  | Radiotherapist (who administrates external beam radiation to cure cancer) |
|  | Psychologist |
|  | Nurse |
|  | Genetist (who give genetic advises) |
|  | Other (traumatologist, thoracic surgeon, etc.) |

**Question 17**

Which is the medical specialist with whom you have had your regular thyroid cancer visits, meaning, which is your regular doctor? (select only one answer)

(random order)

|  | Family doctor |
| --- | --- |
|  | Neck surgeon (either specialist in General Surgery or Otolaryngology) |
|  | Specialist in Nuclear Medicine |
|  | Endocrinologist |
|  | Oncologist |
|  | Radiotherapist |

**Question 18**

Now we will ask you about your personal experience.

We would ask you to rate from 1 to 5 your degree of satisfaction with the treatment you received for your thyroid cancer by the following specialists (5 indicates maximum satisfaction and 1 indicates minimum satisfaction; if any does not apply, please leave it blank)

(random order)

|  | Family doctor |
| --- | --- |
|  | Neck surgeon (either specialist in General Surgery or Otolaryngology) |
|  | Specialist in Nuclear Medicine |
|  | Endocrinologist |
|  | Oncologist |
|  | Specialist in Pathology |
|  | Radiologist |
|  | Radiotherapist |
|  | Psycologist |
|  | Nurse |
|  | Other |

**Question 19**

Rate from 1 to 5 your satisfaction degree with the following aspects of the healthcare centre where your thyroid cancer has been treated and followed-up (5 indicates maximum satisfaction and 1 indicates minimum satisfaction; if any does not apply, please leave it blank)

(random order)

|  | Primary healthcare centre |
| --- | --- |
|  | Outpatient hospital clinic |
|  | Department of Radiology (ultrasound, CT, etc.) |
|  | Department of Nuclear Medicine (radioiodine treatments, scans) |
|  | Department of Surgery (surgical therapy) |
|  | Department of Endocrinology (diagnosis and follow-up of thyroid cancer) |
|  | Department of Oncology (in the case you needed it) |
|  | Department of Radiotherapy (in the case you needed it) |
|  | Department of Clinical Biochemistry (blood tests) |
|  | Department of Pathology (fine-needle aspirations, biopsies) |
|  | Department of Genetics (genetic studies) |

**Question 20**

Rate from 1 to 5 your degree of satisfaction with the information you have received of the different aspects of your treatment and follow-up (5 indicates maximum satisfaction and 1 indicates minimum satisfaction; if there is any that does not correspond, leave it blank)

(random order)

|  | Initial surgical treatment of thyroid cancer |
| --- | --- |
|  | Surgical treatment of relapses of thyroid cancer (nodes, nodules, etc.) |
|  | Radioactive iodine treatment |
|  | Thyroid hormone treatment (Eutirox, Levothroid, Levothyroxine) |
|  | Treatment with tyrosine kinase inhibitors |
|  | External beam radiation treatment |
|  | Psychological and social support treatment |
|  | Blood tests for follow-up (TSH, thyroglobulin, etc.) |
|  | Imaging tests for monitoring (ultrasound, CT, etc.) |
|  | Nuclear medicine tests for follow-up (radioiodine scans) |
|  | Treatment of hypoparathyroidism as a surgical sequel |
|  | Treatment of other sequelae such as dysphonia, lack of saliva, taste, etc. |

Thank you very much for completing this survey.
